# First Report on Co-Occurrence Knockdown Resistance Mutations and Susceptibility to Beta-Cypermethrin in *Anopheles sinensis* from Jiangsu Province, China

**DOI:** 10.1371/journal.pone.0029242

**Published:** 2012-01-17

**Authors:** Wei-Long Tan, Zhong-Ming Wang, Chun-Xiao Li, Hong-Liang Chu, Yan Xu, Yan-De Dong, Zhong-can Wang, Dong-Ya Chen, Hui Liu, Da-Peng Liu, Nannan Liu, Jun Sun, Tongyan Zhao

**Affiliations:** 1 State Key Laboratory of Pathogen and Biosecurity, Beijing Institute of Microbiology and Epidemiology, Beijing, People's Republic of China; 2 Huadong Research Institute for Medicine and Biotechnics, Nanjing, People's Republic of China; 3 Jiangsu Center for Disease Prevention and Control, Nanjing, People's Republic of China; 4 Entomology and Plant Pathology, Auburn University, Auburn, Alabama, United States of America; Universidade Federal de Minas Gerais, Brazil

## Abstract

The increasing prevalence of insecticide resistance in *Anopheles sinensis*, a major vector of malaria in Jiangsu province in eastern China, threatens to compromise the successful use of insecticides in malaria control strategies. It is therefore vital to understand the insecticide resistance status of *An. sinensis* in the region. This study examined the nucleotide diversity of the *para*-sodium channel and knockdown resistance (*kdr*) in five field populations of adult *An. sinensis* mosquitoes collected in Jiangsu province, identifying the L1014F and L1014C substitutions for the first time. Competitive polymerase chain reaction (PCR) amplification of specific allele (cPASA) and polymerase chain reaction restriction fragment length polymorphism (PCR-RFLP) for resistance diagnosis were developed and validated. Comparing the results with direct sequencing revealed that the PCR-RFLP method was more sensitive and specific whereas the cPASA method was more convenient and suitable. The significant positive correlation between *kdr* allele frequency and bioassay-based resistance phenotype demonstrates that the frequency of L1014F and L1014C substitutions in the *kdr* gene provides a useful molecular marker for monitoring beta-cypermethrin resistance in natural populations of *An. sinensis*. Our results point to the L1014F substitution as the key mutation associated with beta-cypermethrin resistance. The high resistance and mutation frequency detected in the five populations also suggest cross-resistance with other pyrethroids may occur in *An. sinensis*, highlighting the need for further surveys to map insecticide resistance in China and the adoption of a rational management of insecticide application for resistance management and mosquito vector control.

## Introduction

The main vectors of malaria in China are *An. sinensis*, *An. anthropophagus*, *An. minimus* and *An. dirus*. Of these, *An. sinensis* is the principal malaria vector in Jiangsu province. The impregnation of bed nets [1]–[3] and indoor residual spraying with pyrethroids [4]–[6] are the primary methods used for vector control in China, but the extensive use of insecticides tends to induce resistance in mosquito populations. Indeed, resistance to pyrethroids and DDT has already been detected in *An. sinensis* and *An. anthropophagus* and is widespread from Jiangsu, Henan, Sichuan and Fujian provinces to the county of Motuo (Tibet) [1]–[2], [7]–[13]. Malaria control programs in Jiangsu would therefore greatly benefit from a better understanding of the status of insecticide resistance in the local *An. sinensis* population and the development of appropriate resistance diagnostic tools.

Pyrethroid insecticides are known to act by modifying the gating kinetics of the *para*-type sodium channels in insect' neurocytes by slowing both the activation and inactivation of the channels [14]. However, modifications in the sodium channel structure such as point mutations or substitutions resulting from single nucleotide polymorphisms [SNP] can dramatically lower sensitivity to DDT and pyrethroids in the sodium channels of the insect's nervous system by reducing or even eliminating the binding affinity of the insecticides to proteins [15], thus diminishing the toxic effects of the insecticides and conferring insecticide resistance [16]. Reduced target-site sensitivity of sodium channels is known to be one of the major mechanisms of pyrethroid resistance and is referred to as knockdown resistance (*kdr*) [17]. *Kdr* was first identified in the house-fly *Musca domestica L*. [18]. Comparisons of partial and complete sequences from 15 susceptible and *kdr* and *kdr*-like resistant housefly strains revealed two point mutations (L1014F and/or M918T) associated with knockdown resistance [19]–[21]. The L1014F substitution has also been reported in many pyrethroid-resistant pest species, including *An. gambiae* [22], *Cx. p. pallens* [23], *Blattella germanica* [20], [24], *Hematobia irritans* [25], *Plutella xylostella* [26], *Leptinotarsa decemlineata* [27], and *Myzus persicae* [28].

Two different *kdr* mutations, L1014F and L1014S, resulting from single nucleotide polymorphisms in the 6^th^ segment of domain II (IIS6) of the *para*-type sodium channels, have been found in the African malaria vector *An. gambiae* [22], [29]. Importantly, both African *kdr* mutations (L1014F and L1014S) were detected in the same individuals in field populations of *An. gambiae* and *An. arabiensis* collected in Uganda by Verhaeghen [30]. Co-occurring *kdr* mutations in the same allele have also been reported in *Cx. p. pallens* mosquitoes in eastern China, where they were linked to pyrethroid resistance [31].

Due to the increasing incidence of knockdown resistance to pyrethroids in pest populations and the disequilibrium of vector control in malaria prevention, better monitoring of knockdown resistance in *An. sinensis* populations is becoming vital. Larval bioassays are generally used for insecticide resistance monitoring in Chinese satellite CDC branches although it is acknowledged that the construction of an insecticide resistance phenotype may differ between the aquatic and adult life stages and that insecticide resistance in larvae is not always transferred through to the adult stage and vice versa. Larvae bioassay was done in this study in order to determine the correlation between the resistance phenotype and knockdown resistance frequencies so as to enhance the work of malaria control in grassroots labs. At present, there is little or no information on the spread of *kdr* mutations in *An. sinensis*. In order to pinpoint the precise genotypic composition and frequency of the *kdr* mutations and link these findings with the insecticide resistance status as defined by bioassays at the end of the intervention period in the five wild populations collected for this study, two approaches were utilized for this research, namely polymerase chain reaction-restriction fragment length polymorphism (PCR-RFLP) and cPASA, to identify the *kdr* genotypes in *An. sinensis* mosquitoes in Jiangsu province.

## Materials and Methods

### Statement of ethical approval

No ethical approval was required as no regulated animals were used in this study. Pre-permission (April 2009–November 2010) was granted for the mosquito observation, adult mosquito collection and field studies in Jiangsu province for this study, which is part of the Infective Diseases Prevention and Cure Project (No: 2008ZX10402). All the field studies on *Anopheles* were authorized by the Committee for Animal Welfare and Animal Ethics in the CDC of Jiangsu province, China (address: 172 Jiangsu Road, Nanjing, Jiangsu province, P. R. China).

### Mosquito strains

**Figure 1:**
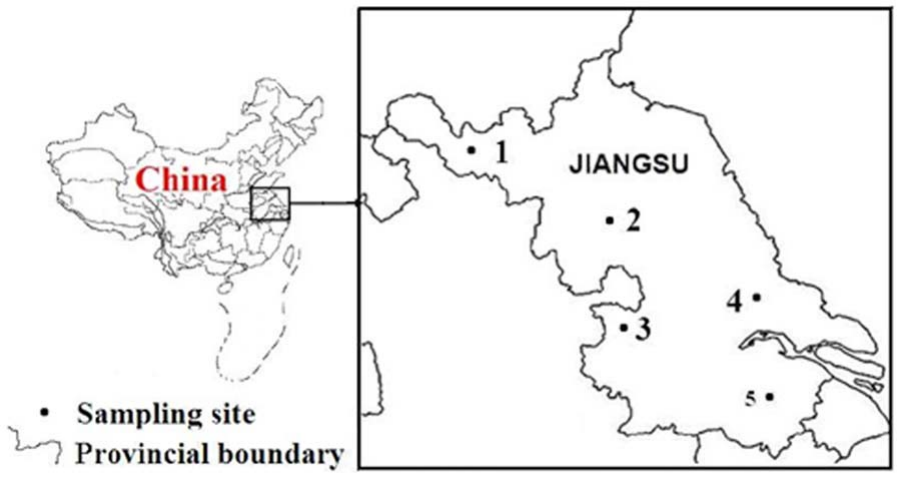
Locations of five blood fed *An. sinensis* field populations. Abbreviations: (1) XZ, XuZhou (34°09′18″N, 117°18′75″E);(2) HY, HuaiYing (33°01′181″N, 118°34′541″E);(3) NJ,NanJing (32°01′18″N,118°28′54″E);(4) CS, ChangShu(31°03′63″N, 119°50′90″E);(5) SZ, SuZhou (31°21′36″N, 120°20′26″E).

A total of five populations of *An. sinensis* adult mosquitoes were collected from July to September 2009 from five sites close to the cities of Xuzhou, Huaiyin, Nanjing, Changshu, and Suzhou. The mosquito populations were named XZ (XuZhou, rural), HY (HuaiYin, rural), NJ (NanJing, rural), CS (ChangShu, rural) and SZ (SuZhou, rural) (Figure 1).

All the mosquitoes were collected in their adult form in rural areas (primarily milk cow cowsheds and pigsties) and transported to insectaria (Insectaria of Huadong Research Institute for Medicine and Biotechnics in Nanjing, Jiangsu, China). The numbers of adults collected were very high from XZ and SZ, 38 adults from HY, 44 adults from NJ, and 49 adults from CS. Most were blood fed when caught. The larvae were supplied with baker's yeast until oviposition occurred. Larvae were kept under the room temperature of 25–27°C and 12 h indoor illumination every day. The density of the larvae was kept at 400–800 larvae/L. After becoming adult, part of each population was stored at −20°C for molecular assay and part for bioassay. One susceptible strain that had been protected from contact with insecticides for 20 years and routinely reared under laboratory conditions was used as a reference for diagnostic tests.

### Specimen identification

Two Anopheles species, *An. sinensis* and *An. anthropophagus*, co-exist in Jiangsu province. But it is difficult to distinguish the two species by dichotomous keys. In order to correlate the dose response with *kdr* genotype frequencies for these specific species, molecular identification by PCR of the 290 specimen samples was carried out using primers designed based on the sequence differences in ribosomal DNA internal transcribed spacer 2 of *An. sinensis* and *An. anthropophagus* [32]. The diagnostic lengths of specific fragments were 425-bp in *An. sinensis* based on the primers UP and PS and 253-bp in *An. anthropophagus* based on the primers UP and PA (Table 1).

**Table 1:** PCR primers used in this study.

Name	Sequence (5′→3′)	
D1	AAR YTN GCN AAR TCT TGG CC	(Martinez-Torres, 1998)
Dg2	GCD ATY TTR TTN GTN TCR TTR TC	(Martinez-Torres, 1998)
CN1	TGG CCN ACG CTG AAY TTA CTC	
CN2	CCG AAATTG GAC AAA AGC AAA G	
CP1	TGATCGTGTTTCGCGTGCTG	
CP2	GCGTCTCGTTATCCGCCGTT	
CD1	TGATCGTGTTTCGCGTGCTG	
CD2	GTCTCGTTATCCGCCGTTGG	
Cgd3	CCCGGTGGTAATTGGAAACTTG	
Cgd4	TGCGGTGGTAATTGGAAACTTT	
Cgd5	TGCGGTGGTAATTGGAAACTGT	
UP	CCA TGACGTACACA TACTTG	(Ma *et al*. 1998)
PA	GCTCCA TCTACACACA GCGT	(Ma et al. 1998)
PS	GTTGTCCA GCCCGCTAACA T	(Ma *et al*. 1998)
EP1	gcggtcccaaaagggtcagtTAGCCACTGTGGTAATTGGAAgCT	
EP2	gcggtcccaaaagggtcagtTGGTGCAGAGAGCGATGATG	
EP3	gcggtcccaaaagggtcagtGGAGTGGATCGAATCAATGTGG	
EP4	gcggtcccaaaagggtcagtTGTCGTCCTGCAGTTACTCAtCAC	

### Bioassays

In order to correlate the *kdr* genotypes with their resistance phenotypic outcomes, larvae bioassays were performed on the six populations. Beta-cypermethrin powder (Tianjin Pesticide Co. Ltd, China), the mostly widely used insecticide in the malaria control effort in Jiangsu province, was completely dissolved in acetone. The stock solution, with a final concentration of 20 ppm, was stored at 4°C for less than 2 months. The stock solution was diluted in seven concentrations along a gradient from multiproportion. Bioassays were made using late third and early fourth instar larvae. Batches of ninety larvae per concentration were respectively put into three parallel cups. Each cup contained 199 ml distilled water and 1 ml of insecticide solution and this provided a range from 0 to 100% mortality. Mortality was recorded after a 24-hour exposure. The temperature was maintained at 26±1°C during the bioassay. The mortality of a control group exposed to water and 1 ml of acetone never exceeded 4%.

### Partial sequencing of the sodium channel cDNA

The IIS4-IIS6 coding region sequences of the sodium channel gene were sequenced from a susceptible strain (SS) and three resistant wild specimens from the HY (HuaiYin) strain. Total RNA was extracted with Trizol (Invitrogen, USA) from a single mosquito specimen in each of the five samples. The first-strand cDNA synthesis was performed using the SuperScript™ III First-Strand Synthesis System for RT-PCR (Invitrogen, USA) and an oligo (dT) adapter primer. Two steps of PCR were performed. Primary PCR on the single-stranded cDNA was carried out with the degenerate primers D1 and Dg2 [22]. One unit of Taq polymerase (TaKaRa) in buffer (supplied by the manufacturer), 200 ng of each primer and 0.2 mM dNTP were used in a 25-µl total volume PCR reaction consisting of 1 cycle of 94°C for 3 min and 35 cycles at 94°C for 1 min, 50°C for 30 sec, 72°C for 1 min and a final extension step at 72°C for 7 min. The secondary step of PCR was then carried out based on the primary product with nested inner primers of CN1 and CN2 (Table 1) based on the partial sequencing of *para*-type sodium channel of *M. domestica* [21], *B. germanica* [20], *An. gambiae* [22] and *Cx. P. pallens* [23]. The amplified fragment (Genbank accession number: JN002364) was then recovered by Wizard PCR preps DNA Kit and used as a template for direct sequencing with the automated ABI PRISM Dye Terminator Cycle Sequencing Kit.

### Intron sequence determination

Genomic DNA of the five strains, XZ, SZ, NJ, CS and HY, was respectively extracted from a single mosquito of each using Universal Genomic DNA Extraction Kit Ver 3.0 (TaKaRa) according to the method of Collins [33]. The genomic region containing the intron sequence of interest, which was located 2 bp downstream of the *kdr* mutation, was PCR amplified on 10–50 ng of genomic DNA using primers CP1 and CP2 (Table 1). One unit of KOD plus polymerase (TOYOBO) and 100 ng of each primer were used in a 50-µl total PCR volume. Amplification was performed as follows: 1 cycle of 94°C for 3 min and 35 cycles at 94°C for 30 sec , 54°C for 30 sec, and 68°C for 1 min with a final extension step at 68°C for 7 min. After sequencing, one intron manifested its size.

### Assay for kdr genotype

**Figure 2:**
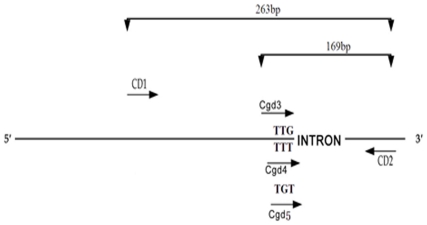
Schematic diagram of cPASA strategy for detecting L1014F and L1014C substitutions and predicting the size of PCR products in the *para* sodium channel gene in *An. sinensis*. CD1-CD2 and Cdg3-Cdg5 indicate PCR primers whose sequences are reported in Table 1. Primer pair Cdg3 and CD2 amplifies a 169-bp fragment for the wildtype susceptible allele (for codon TTG). Primer pair Cdg4 and CD2 yields a 169-bp fragment for resistant L1014F allele (codon TTT). Similarly, primer pair Cdg5 and CD2 leads to amplification of a 169-bp fragment diagnostic to the L1014C resistant allele (codon TGT). The primer pair CD1 and CD2 is two allele-nonspecific outer primers.

Based on the sequence data for *An. sinensis* and the methods of competitive PASA (cPASA) described by Jamroz *et al*. [34], Martinez-Torres *et al*. [22], Zhang *et al*. [35] and Song *et al.* [36], with some modifications, a test method involving three PCR reactions for each specimen was developed to diagnose the *kdr* genotypes (Figure 2). The three PCR reactions were almost the same except that one contained a sense-specific primer (cdg3) ending with the two bases “TG” in the 3′end position to detect the susceptible codon “TTG”, the second contained a sense-specific primer (cdg4) ending with the two bases “TT”in the 3′end position to detect the mutation codon “TTT”, and the third reaction contained the third sense-specific primer (cdg5) ending with the two bases “GT”in the 3′end position to detect the mutation codon “TGT”. Thus, a total of three allele-specific inner primers were designed: sensitive sense-primer Cgd3 and two resistant sense-primers Cgd4 and Cgd5 (Table 1). Two additional nonspecific outer primers were based on the sequence immediately downstream from the mutation site and one sense primer upstream of the site, thus bracketing the mutation points. The two allele-nonspecific outer primers were sense primer CD1 and anti-sense primer CD2 (Table 1).

A PCR diagnostic test was performed in accordance with the standard procedure using a total volume of 50 µl, consisting of 10× Buffer 5 µl, 80–100 ng genomic DNA 4 µl, 50 mM MgCl_2_ 2 µl, 10 mM each dNTP 2 µl ,1 µl KOD plus polymerase, 2 µl primers (1 µl of each primer), 34 µl dH_2_O. For each template from an individual strain, there were three PCR reactions, of which the first reaction included the primers CD1, Cgd3 and CD2, the second CD1, Cgd4 and CD2, and the third CD1, Cgd5 and CD2. The PCR conditions were one cycle of 93°C for 4 min, then 35 cycles of 94°C for 1 min, 55°C for 30 sec and 68°C for 1 min, followed by one cycle of 68°C for 7 min. PCR products were checked by electrophoresis on 1.2% agarose gel in TAE buffer. The resulting bands were visualized by ethidium bromide staining. The diagnostic PCR products for the *kdr* alleles were 169-bp and those for the allele-nonspecific outer primers were 263-bp.

### PCR-RFLP protocol for *kdr* genotype discrimination and direct sequencing of the *kdr* gene

**Figure 3:**
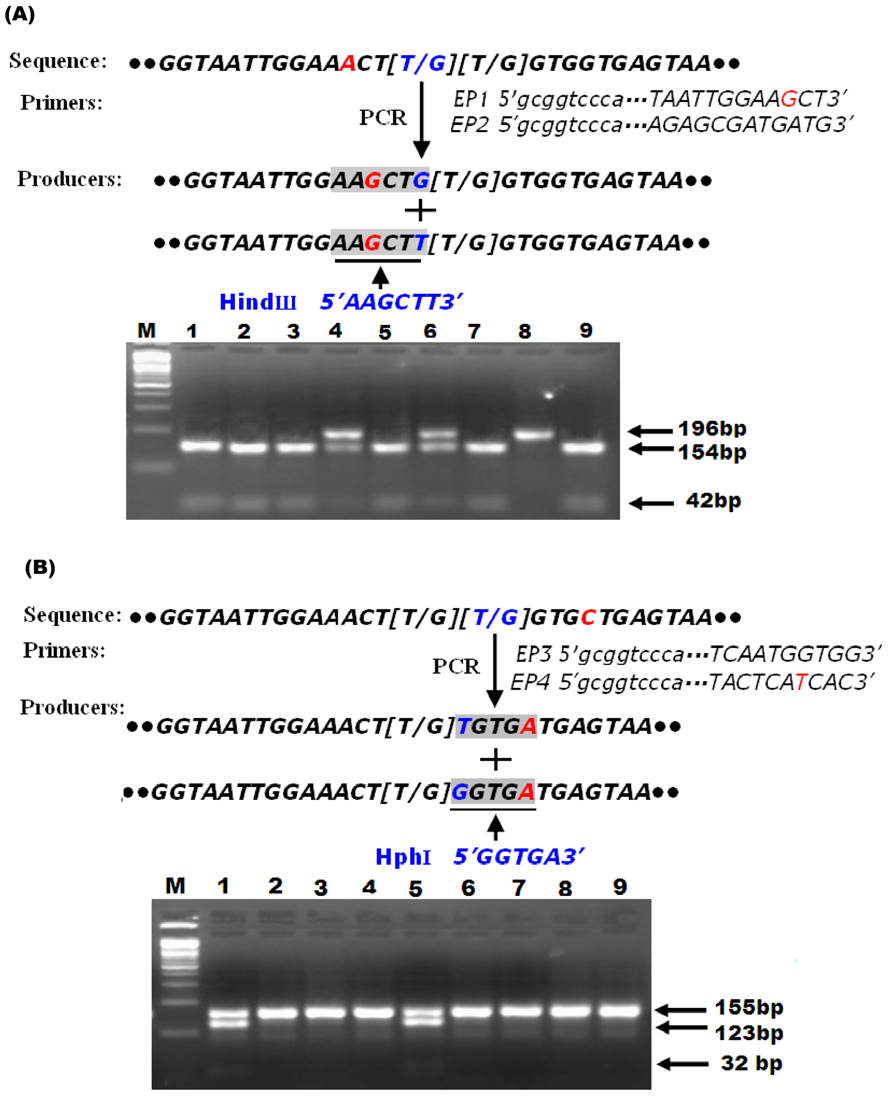
Schematic diagram of PCR-RFLP strategy for detecting L1014F and L1014C substitutions and predicting the size of PCR-RFLP products in the *para* sodium channel gene in *An. sinensis*. **A**: A“A”to “G” substitution at the *EP1*-primer causes the formation of a diagnostic HindIII recognition site (gray shade) at position 42-bp of the PCR fragment.“M” is a marker. The substitution can be inferred from the appearance of the agarose gel electrophoresis: “1,2,3,5,7,9” are (T/T), “4,6” are (T/G), “8” is (G/G). **B**:A“C”to “T” substitution at the *EP4*-primer causes the formation of a diagnostic HphI recognition site (gray shade) at position 32-bp of the PCR fragment.“M” is a marker. The substitution can be inferred from the appearance of the agarose gel electrophoresis: “2,3,4,6,7,8,9” are (T/T), “1,5” are (T/G).

Two approaches, namely PCR-RFLP (Figure 3) and direct sequencing, were used to further confirm the *kdr* genotype. We developed a PCR-RFLP method for genotyping both mutations using two PCR reactions followed by two restriction digests and agarose gel electrophoresis. Two sets of primers were used to identify both mutation sites. In this study, the point mutation in L1014 in domain IIS4- IIS6 of the sodium channel gene was present in the form T (T/G)(T/G). The first PCR reaction was used to detect the first “T/G” based on the primers EP1and EP2. An “A” to “G” mismatch at the EP1-primer (forward) at the 3rd base position from 3′ end terminus (shown in small letter in primer sequence) was designed. The terminal AAgCT-3′ sequence with the first (T/G) may create a recognition site for the restriction enzyme HindIII AAGCTT at the 42-bp position in the PCR fragment. The second PCR reaction was used to detect the second “T/G” based on the primers EP3 and EP4. A “C” to “T” mismatch at the EP4-primer (reverse) at the 4rd base position from 3′ end terminus (shown in small letter in primer sequence) was designed. The terminal tCAC-3′ sequence with the second (T/G) may create a recognition site for the restriction enzyme HphIGGTGA at the 32-bp position of the PCR fragment. Both PCR reactions were done with the previously described PCR conditions, briefly 10×Taq buffer 1.5 µl, 1.2 µl MgCl_2_, 0.1 µl Taq polymerase (TaKaRa), 0.3 µl dNTP mixture, 0.4 µl primers (each primer 0.2 µl), 0.5 µl template DNA and 11 µl dH_2_O in a 15-µl total volume, PCR reaction consisting of 1 cycle of 94°C for 3 min, 35 cycles at 94°C for 30 sec, 66°C for 30 sec, 72°C for 60 sec. Then the PCR product was stored on ice at −0.4°C until the final stage in the process, which consisted of another 20 cycles at 95°C for 30 sec and 68°C for 60 sec and a final extension step at 72°C for 6 min. The restriction digest reaction involved 0.2 µl (10 U/ml) enzyme and 15 µl PCR reactant in a 20-µl total digest volume. The electrophoresis was performed with 4% agarose at 150 V for 90 min.

## Results

### Species identification and beta-cypermethrin resistance of Anopheles populations

Two hundred and ninety specimens of *Anopheles* complex mosquitoes were collected during the survey and tested with a Polymerase Chain Reaction (PCR) adapted from Ma *et al.* [32] to distinguish the *An. sinensis* and *An. anthropophagus*. PCR assay gave the 452-bp species-specific fragments of *An. sinensis*, no amplification of the 253-bp fragment signifying the absence of *An. anthropophagus*. All the tested mosquitoes were therefore deemed to be *An. sinensis* in agreement with the known geographic distribution of species within the *An. anthropophagus* complex in Jiangsu province, eastern China. In the bioassays, the five populations all showed high resistance to beta-cypermethrin (Table 2). Resistance ratios of LC_50_ for the five populations were found to range from 700 to 2100-fold, much higher than the 3.5- to 5-fold reported for *An. sinensis* mosquitoes from Liaoning province [37].

**Table 2:** Values of 50% lethal concentration (LC_50_) for the five *An. sinensis* resistant field populations compared to the susceptible lab strain in response to beta-cypermethrin.

Mosquito Population	LC50(ppm)	95% confidence interval	R/S*
SS	0.001	0.0002–0.001	1
XZ	0.9	0.3–3.5	900
HY	1.3	0.2–11	1300
NJ	1.7	0.4–3.3	1700
CS	2.1	1.3–3.1	2100
SZ	0.7	0.46–1.0	700

*R/S is the ratios of LC50 of the test population to the SS population.

### Partial sequencing of the sodium channel cDNA

**Figure 4:**
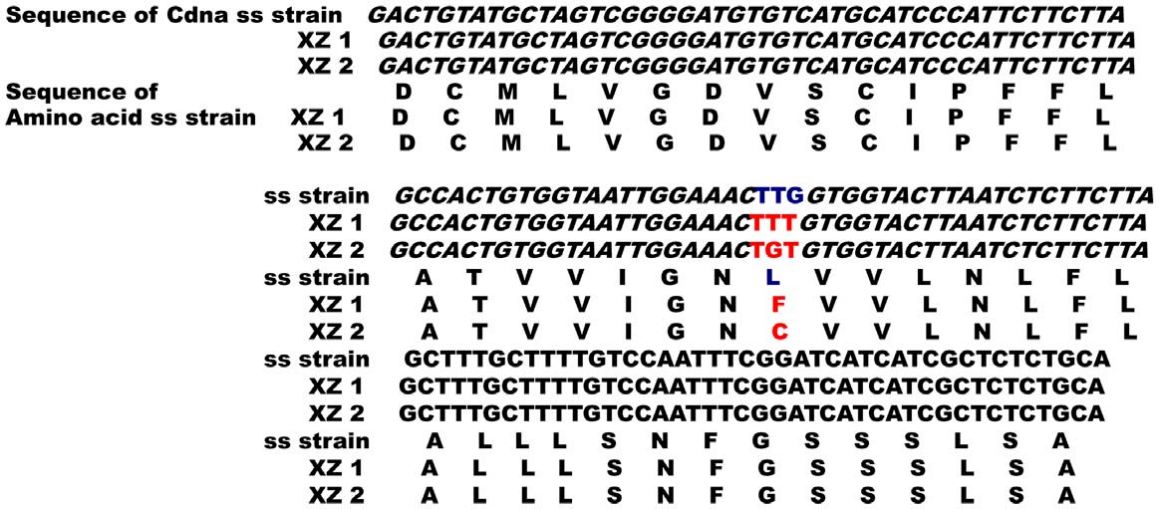
The sequences of cDNA and amino acids from the susceptible SS strain and resistant XZ populations. The sequences XZ1 and XZ2 come from cloning *An. sinensis* individuals from the XZ population. The sequence of the SS strain amino acid was translated from the DNA sequence of the *An. sinensis* SS strains. XZ1 and XZ2 amino acid sequences were translated from XZ cDNA sequences by cloning.

A 357-bp cDNA sequence of individual mosquitoes was amplified from the susceptible strain and the wild resistant strain from XZ (XuZhou). The nucleotide sequences were the same except a difference at position 1014, where TTG (Leu) in the SS strain sequence was replaced with TTT (Phe) or TGT (Cys) in the XZ strain sequences at amino acid 1014 (Figure 4).

### Intron sequence determination

Blast comparison of the partial cDNA sequence of the sodium channel gene and comparing the results with those given on the website (http://genome.ucsc.edu/cgi-bin/hgBlat), the results showed that the intron was located in the domain II region, just at the conserved positions for *Cx. P. pallens* [22]. One intron was located downstream of the *kdr* mutation and the knowledge of its sequence length was necessary in order to discern the size of the allele using the PASA test. The PCR products amplified by CP1 and CP2 from individual genomic DNA extracted from the five strains were sequenced. No polymorphism was found in this intron after comparing several specimens.

### Optimization of *kdr* mutation diagnostic assays

**Figure 5:**
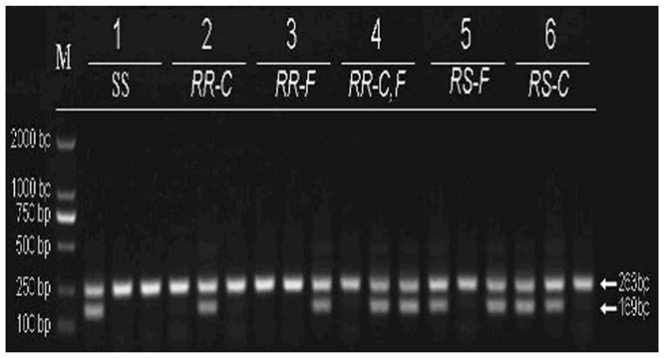
Schematic diagram of CPASA detection gel for L1014F and L1014C sodium channel substitutions in *An. sinensis*. The allele-specific k*dr* diagnostic test developed by Martinez-Torres et al. [22] was adapted to detect *kdr* genotypes of individuals from 6 typical wild specimens. M: marker. The 263-bp fragment was the out control and the 169-bp fragment was the *kdr* specific allele. Genotype results:specimen 1:TTG/TTG;specimen 2:TGT/TGT;specimen 3:TTT/TTT;specimen 4:TGT/TTT;specimen 5:TTG/TTT;specimen 6:TTG/TGT.

After optimization, the PCR diagnostic test was capable of discerning the *kdr* homozygous (RR) and heterozygous (RS) genotypes for the L1014F and L1014C substitutions in the sodium channel a-subunit gene *para*. The product amplified by two non-specific outer primers CD1 and CD2 was 263-bp. The product amplified by three specific paired-primers was 169-bp (Figure 5).

### Results of PCR-RFLP and direct sequencing

After the first PCR reaction based on the primers EP1 and EP2 and digested with HindIII, the presence of the PCR products in the electrophoresis could be identified as *kdr* genotypes. A sequence with G/G in the first (T/G) position could be inferred by the appearance of a single 196-bp fragment, T/T was inferred by the appearance of a 154-bp and a 42-bp fragment and G/T was inferred by the appearance of three fragments, of 196-bp, 154-bp and 42-bp. In the same way, the second (T/G) involving (T/T), (G/G) and (G/T), was inferred by the three fragments of 155-bp, 123-bp and 32-bp, respectively (Figure 3). Direct sequencing was used to validate the genotyping results of cPASA and PCR-RFLP. The sensitivity and specificity of the two genotype methods was found to exceed 94% in all cases (Table 3).

**Table 3:** Sensitivity of the cPASA and PCR-RFLP methods in reference to allele sequence data for identification of *kdr* genotypes in *Anopheles sinensis*.

	Detection methods		
	Sequencing(n = 50)	cPASA(n = 50)	PCR-RFLP(n = 50)
Genotype frequency			
TTG/TTG	0.00	0.00	0.00
TTT/TTT	68	72	68
TGT/TGT	4.0	2.0	6.0
TTG/TTT	2.0	2.0	4.0
TTT/TGT	20	22	20
TTG/TGT	6.0	2.0	2.0
Sensitivity (%)		94	96
Specificity (%)		94	96

The 50 *Anopheles sinensis* individuals used in this assay were from the XZ (XuZhou) population.

### Distribution of *kdr* allele frequencies in natural populations from eastern China

A total of five field populations, 290 specimens collected from rural locations close to the cities of XZ, SZ, CS, NJ and HY, plus the susceptible SS strain were tested with cPASA. Table 4 summarized the results of the cPASA assays. Based on the presence or absence of the *kdr* alleles, individual mosquitoes were genotyped as homozygous susceptible (SS), homozygous resistant (RR), or heterozygous (RS). No L1014C (TTG/TCG) genotype was found in any of the mosquito samples tested. Only one specimen from XZ (XuZhou) was found to be homozygous susceptible (SS). None of the two resistant alleles were present in any of the specimens from the laboratory reference strain. PCR assays revealed clear differences in overall *kdr* allelic frequency between the resistant and susceptible strains. All the SS strain specimens were homozygous susceptible but *kdr* frequencies in the wild resistant strains ranged from 16% to 84%. The *kdr* alleles existed mainly in the *kdr*-F/F and *kdr*-F/C genotype, the resistant homozygous form, and only a small portion (1–4%) of the mosquitoes possessed *kdr*-C homozygous (RR-C) *kdr* alleles, with 3–5% of the *kdr*-C and *kdr*-F heterozygous (RS-C and RS-F) *kdr* genotype in the five populations. *Kdr* allelic frequency ranged from 74% to 84% on the *kdr*-F, and 16% to 24% on *kdr*-C. The genotype frequency of RR-F and RR-F/C ranged from 51% to 69% and 29% to 47%, respectively.

**Table 4:** Frequencies (in percentages) of *kdr* alleles and genotypes in relation to the five *An. sinensis* populations from east-China monitored by cPASA.

Populations	SS	XZ	HY	NJ	CS	XZ
Sample size (n)	50	84	38	44	49	75
Frequency of *kdr* allele (%)						
TTG (L1014)	100	5	1	1	1	0
TTT (1014F)	0	75	75	77	76	84
TGT (1014C)	0	20	24	22	23	16
Frequency of *kdr* genotype (%)						
L/L (TTG/TTG)	100	1	0	0	0	0
L/F (TTG/TTT)	0	5	3	2	2	0
L/C (TTG/TGT)	0	4	0	0	0	0
F/F (TTT/TTT)	0	59	53	61	51	69
C/C (TGT/TGT)	0	4	3	3	0	1
F/C (TTT/TGT)	0	29	43	30	47	29

### Frequency of L1014F and L1014C substitutions in response to beta-cypermethrin

**Figure 6:**
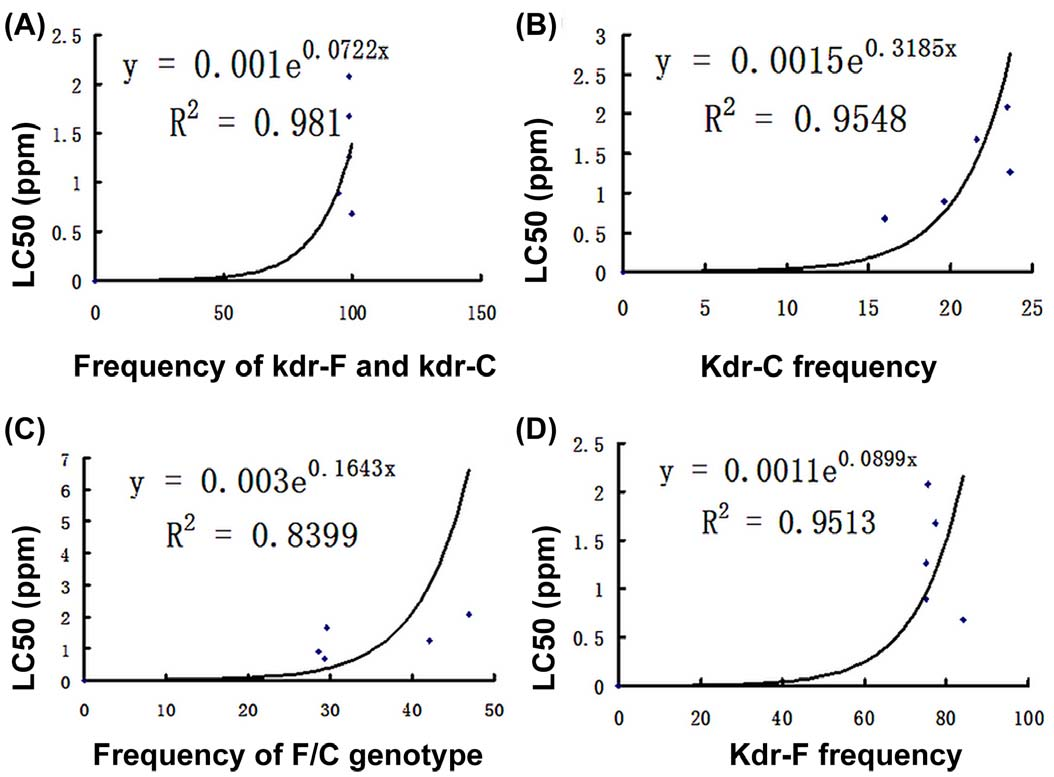
Correlation between LC_50_ and percentage frequencies for *kdr* alleles and genotypes. (A), (B), (C), (D): exponential correlation relationship between frequencies of *kdr*-F and *kdr*-C, *kdr*-C, F/C genotype, *kdr*-F genotype and the 50% lethal concentration of the five populations against beta-cypermethrin.

Spearman's rank correlation analysis based on the correlate analysis showed significant correlations between the LC_50_ and the *kdr* allele frequencies of *kdr*-F and *kdr*-C (p≤0.01). Regression analysis revealed a significant correlation between LC_50_ estimates and the frequency of *kdr*-(F+C)(R^2^ = 0.981), *kdr*-C (R^2^ = 0.9548), *kdr*-F (R^2^ = 0.9513) and F/C genotype (R^2^ = 0.8399) (Figure 6). In addition, the frequencies of the RR-F/C (TTT/TGT) genotype in the HY and CS populations (42% and 47%, respectively) were higher than that of the populations from SZ and XZ.

### Hardy-Weinberg equilibrium test

The Hardy-Weinberg equilibrium test was conducted for analysis of the *kdr* genotype frequencies in each population with the T-paired test. The unbiased estimates of *P*-value of the five populations were 1.0, 0.9, 1.0, 1.0, and 0.9 for XZ, HY, NJ, CS and SZ, respectively. The results showed that there were no significant differences between the expected values and the observed values either in genic or in genotypic differentiation. The five populations were therefore all deemed to be present in genetic equilibrium (Table 5).

**Table 5:** *Hardy-Weinberg* equilibrium tests on *kdr* genotype frequencies.

Genotype Frequency		SS (L/L)		RR (F/F)		RR (C/C)		RS (L/F)		RS (L/C)		RR (F/C)		t	*P*
population	Sample Size (n)	Exp	Obs	Exp	Obs	Exp	Obs	Exp	Obs	Exp	Obs	Exp	Obs	/	2-tailed
XZ	84	0.003	0.01	0.6	0.6	0.04	0.04	0.084	0.05	0.02	0.04	0.3	0.3	−0.009	1.0
HY	38	0.0002	0.00	0.6	0.5	0.06	0.00	0.02	0.03	0.006	0.03	0.36	0.4	−0.09	0.9
NJ	44	0.0001	0.00	0.6	0.6	0.05	0.03	0.02	0.02	0.005	0.00	0.3	0.3	0.01	1.0
CS	49	0.0001	0.00	0.6	0.5	0.06	0.00	0.02	0.02	0.005	0.00	0.4	0.5	−0.00	1.0
SZ	75	0.00	0.00	0.7	0.7	0.03	0.00	0.00	0.00	0.00	0.01	0.3	0. 3	0.1	0.9

T-Paired test: *P* (2-tailed) = 1.0(XZ), 0.9 (HY), 1.0(NJ), 1.0 (CS), 0.900(SZ).

## Discussion

Knockdown resistance (*kdr*) is a type of target-site resistance arising from point mutations in the sodium channel genes of the insect nervous system and is known to confer cross-resistance to DDT and pyrethroids [38]. The resistance of *An. sinensis* against pyrethroids and DDT has been increasing rapidly in China, but *kdr* mutations had not previously been detected. To investigate whether this mechanism was general in *An. sinensis*, the partial sequences of the *para*-type sodium channel genes from various *An. sinensis* field populations collected from five sites in Jiangsu province in eastern China were examined. The sequence observed in the lab SS strains of *An. sinensis* was not consistent with that in the other mosquito species for one codon: the silent replacement of TTA (Leu) by TTG (Leu) at amino acid 1014. After comparing the sequences in the field populations and the SS strain, as reported in *An. gambiae, Cx. p. pallens* and *Cx. p. quinquefasciatus*, two types of molecular mutations were found at L1014 alleles in the Xuzhou (XZ) population, in which the resistance level was about 900 fold that of the susceptible SS strain to Beta-cypermethrin. The first point mutation, with TTG (Leu) being replaced with TTT (Phe), accords with prior reports of the *kdr* mutation in *An. gambiae* [39], *Cx. p. pallens* [36], *Cx. p. quinquefasciatus* [40]. The second, TTG (Leu) being replaced with TGT (Cys), is a new mutation.

The *Leu/Phe* substitution has been implicated in the development of pyrethroid resistance in several mosquito species, including *Cx. p. pallens* [41], [42] and *Cx. p. quinquefasciatus* [43]. Previous electrophysiological studies have shown that the *kdr* substitution of L1014F on domain II S6 promotes closed-state inactivation so 70–80% of the sodium channels never open [44], thus the *Leu/Phe* substitution is thought to directly cause the heightening of the insect's resistance based on the results of the bioassay. Since all the tested field populations showed high resistance to beta-cypermethrin, the relationship between L1014F substitution frequency and mosquito survival when challenged with beta-cypermethrin was analyzed in this study. The L1014F substitution not only showed a strong positive correlation with LC_50_ (R^2^ = 0.9519) but also a significantly high frequency (75% to 84%), corresponding to the resistance level against beta-cypermethrin in all the field populations (700 to 2100-fold). This finding therefore supports previous studies that suggested that the L1014F substitution is the key mutation responsible of beta-cypermethrin resistance. An alternative substitution of leucine to cysteine (Leu to Cys) in the same location showed a relative low frequency within populations; in spite of its strong positive correlation with LC_50_, this *Leu/Cys* substitution is unlikely to play an important role in beta-cypermethrin resistance. Martinez-Torres [22] reported that L1014S substitution in one strain showed a slight increase in resistance to pyrethroids but greatly increased DDT resistance. It seems reasonable to suppose, therefore, that the L1014C substitution may be responsible for resistance to other insecticides within the same family of pyrethroids and DDT. Based on the positive correlation between LC_50_ and the total frequency of *kdr*-F and *kdr*-C (R^2^ = 0.981), taken together with the results of previous studies on other mosquito species, we consider that *kdr* mutation screening offers an excellent molecular marker for pyrethroid resistance monitoring in *An. sinensis*.

Sensitive detection of the mutations associated with knockdown resistance is a prerequisite for resistance management strategies aimed at prolonging insecticide lifetime while maintaining sufficient insect control. A number of methods, for example Allele-specific PCR (AS-PCR), Hot Oligonucleotide Ligation Assay (HOLA), TaqMan probe and PCR-RFLP, have been utilized to detect *kdr* mutations in several species [45], [46]. In this study, based on the specific mutation types identified in *An. sinensis*, we developed CPASA and PCR-RFLP methods to detect the two forms of *kdr* alleles and compared the sensitivity and specificity of the two methods to that obtained using direct sequencing. Although some researchers had reported that the CPASA method could lead to unreliable results [31], [45], our results in the present study indicated that the sensitivity and specificity of this method were relatively high. Considering quick results and low costs, CPASA seems to be a good candidate for automation with microplates and robotic workstations for high throughput. In contrast, PCR-RFLP is less widely used for genotyping *kdr* alleles because this approach suffers from severe limitations in experiments that lack a digestion pattern and is not suitable for large-scale point mutation screening for low mutation frequency conditions, especially when the relatively high capital expenditure and running costs become a major consideration. However, implementing the PCR-based approach followed by RFLP for allele identification is robust, simple to perform, and easy to interpret, which makes it eminently suitable for use in reference laboratories. In this study, PCR-RFLP was used to further confirm the results obtained from CPASA under strict PCR and agarose gel electrophoresis conditions only when substitutions were used to supply digestion patterns in the primers. The results confirmed that PCR-RFLP was higher in both sensitivity and specificity than CPASA for the alleles genotyped here.

It is important to obtain a baseline level for insecticide sensitivity on both the local and regional scales so that resistance management can be adjusted appropriately to local conditions. Although the results reported here refer to relatively few *An. sinensis* samples from a very large geographical range, they represent a first effort to analyze the overall distribution of the L1014F and L1014C substitutions in Jiangsu province in East China, where both molecular forms of these species co-occur over most of their range of distribution. The results of the Hardy-Weinberg equilibrium test showed that all the five field populations used for this study were presently in Hardy-Weinberg equilibrium. Our results indicate that there is indeed an increased selective pressure due to the use of pyrethroid insecticide in this region. Since larvae of *An. sinensis* are most likely living in the rice paddies in the province, insect control activities such as the large-scale use of pyrethroid insecticides for agricultural purposes, and possibly for domestic protection, may be a major factor contributing to the increasing insecticide resistance of *An. sinensis*. Although beta-cypermethrin is not widely used for agricultural purposes, the high resistance against it exhibited by mosquitoes throughout the region suggests that cross-resistance with other pyrethroids may be occurring in *An. sinensis*, so rational management of insecticide applications should be carried out in the future in order to minimize the development of resistance and thus better support vector control efforts.
